# ‘The child that I left behind’: memory, trauma, and the reconstruction of childhood in Nakba narratives

**DOI:** 10.3389/fpsyg.2025.1656549

**Published:** 2025-11-05

**Authors:** Rawan Nasser

**Affiliations:** The Minerva Center for Human Rights, Hebrew University of Jerusalem, Jerusalem, Israel

**Keywords:** childhood memories, trauma, settler colonialism, unchilding, Nakba, decolonial knowledge

## Abstract

**Introduction:**

This article analyzes Palestinian refugee childhood memories, focusing on how displacement and survival intersect within the context of ongoing settler-colonial violence. Challenging conventional Western trauma frameworks that view trauma as discrete, time-bounded events amenable to therapeutic resolution, this research conceptualizes Palestinian children’s experiences as sociogenic trauma emerging from colonial structures rather than individual pathology.

**Methods:**

Drawing on 34 interviews with Palestinian refugees from Lydda who experienced the 1948 Nakba as children or were born shortly after, the study uses the child as method framework to analyze childhood memories as complex and dynamic sites where trauma and adaptive survival mechanisms coexist and shape individual and collective experiences.

**Results:**

Findings reveal systematic processes of “unchilding”—the deliberate eviction of Palestinian children from childhood through invisibilization, dehumanization, and forced premature maturation—alongside survival strategies such as selective sensory silencing and strategic memory suppression. The study demonstrates how Palestinian refugees mobilize childhood memories to position themselves within ongoing displacement, deploying childhood as a cultural-political category to navigate present conditions of ongoing Nakba and resistance.

**Discussion:**

This study contributes to scholarship that centers Palestinian perspectives by illuminating how childhood memories function as sites of resistance that protect Palestinian knowledge from appropriation. It calls for fundamental changes in academic and professional practice, advocating approaches that honor Palestinian epistemologies while challenging Western frameworks’ claims to universality in understanding trauma and survival.

## Introduction

Ali, who was 8 years old during the Nakba in 1948, came to our interview in Ramallah carrying an old history book documenting Lydda, his city of origin, along with family photographs and stories about the city. Rather than beginning the conversation with predetermined lines of inquiry about historiography, I invited him to reflect on his childhood experiences within the specific temporal and spatial context of Lydda. After a thoughtful pause, he shared the following:

"For most 8-year-olds, what are the biggest worries? Playing outside with friends, going to school, doing what children usually do… But for me, being 8 years old meant suddenly having to bring water for my little sisters, walking for hours under the hot sun—barefoot most of the time—just to find something to eat…witnessing people dying along the roads… the fear of death that controlled me. That's what I remember from my childhood."

Despite not being explicitly asked about the Nakba and being encouraged to recall childhood experiences preceding displacement, Ali did not articulate any pre-Nakba memories of life in Lydda. When prompted to reflect on this temporal selectivity, Ali responded:

"These memories are stronger, perhaps because this pain has never been healed. Perhaps because since that time I grew up and was never allowed to return to that child again… since the Nakba, I couldn't remember anything beyond the loss of our homes and land."

His immediate shift to recounting experiences of displacement, even when prompted to reflect on his pre-Nakba childhood, raises critical questions about the function of childhood memories in contexts of settler colonial violence not simply as static repositories of the past, but as dynamic sites where meanings are continually negotiated and redefined. This article explores such trauma through the memories of Palestinian children who, like Ali, experienced the Nakba as children or were born in its immediate aftermath. Rather than approaching these memories through conventional Western trauma frameworks—which conceptualize trauma as discrete, time-bounded events amenable to therapeutic resolution—this research employs a decolonial approach that challenges Western knowledge systems while centering Palestinian epistemologies. The study investigates how childhood memories are shaped within ongoing settler-colonial violence, how these memories serve as sites of epistemological refusal, and how they reveal sophisticated survival strategies that Western frameworks have rendered invisible or pathological.

Drawing on Burman’s (2017) child as method framework, this research analyzes how Palestinian refugees mobilize childhood memories and tropes to position themselves within current political contexts of ongoing displacement. Rather than treating these memories as direct windows into past experience, child as method examines how narrators deploy childhood as a cultural-political category to navigate present conditions of exile and resistance. This approach reveals how the ‘child’ functions not merely as a subject of memory but as a method for understanding how settler-colonial power operates across generations, targeting Palestinian social reproduction through the systematic disruption of childhood itself.

This research contributes to decolonial scholarship by showing how Palestinian childhood memories enact what Simpson (2007) terms ethnographic refusal—the deliberate withholding of experiences from Western academic consumption to assert Palestinian narrative sovereignty. By centering Palestinian epistemologies and challenging Western universalism, this study reveals childhood memories as complex knowledge systems that resist settler-colonial erasure while safeguarding what is sacred from colonial appropriation.

### The Nakba (1948 catastrophe)

Meaning “catastrophe” in Arabic, the term Nakba refers to the events surrounding the establishment of Israel in Palestine in 1948. The Nakba has been described by both Palestinian and Israeli historians as the destruction of historic Palestine (Pappé, 2006; Masalha, 2018). It resulted in the forced displacement of at least 80 percent (over 750,000) of Palestinians living in areas that became Israel, encompassing 77 percent of historic Palestine. These individuals were either internally displaced or expelled from their homeland. More than 500 Palestinian villages were destroyed, creating substantial refugee populations across neighboring Arab states (Pappé, 2007; Sa'di and Abu-Lughod, 2007). Scholars have characterized the Nakba as a multifaceted catastrophe, encompassing the mass uprooting of people from their homeland, the destruction of social structures that had endured for centuries, and the frustration of Palestinian national aspirations (Sa'di, 2008; Galtung, 2012). At its core, the Nakba was marked by dispersion, helplessness, violence, and humiliation—symbolizing what many Palestinians experienced as unexpected and unstoppable destruction that left communities in a state of political, economic, and psychological disarray (Sa'di and Abu-Lughod, 2007).

The Nakba represents a foundational trauma in Palestinian collective memory. Palestinians have never recovered from the material and psychic reality of the 1948 catastrophe: for every household there is a Nakba story, or each refugee an uprooted home. The ramifications of the Nakba continue to shape Palestinian society across generations, as millions are still born into refugee status and continue to languish in refugee camps. Palestinian scholars have conceptualized this situation into what is termed the “ongoing Nakba”—a present continuous condition that defines contemporary Palestinian existence under continued displacement, occupation, and dispossession (Masalha, 2012).

### The Nakba as continuous trauma

The ongoing nature of the Nakba distinguishes it from other global catastrophes by highlighting the continuous process of displacement and dispossession rather than framing it as a singular historical event marked by immediate death tolls (Abu-Sitta, 1998; Masalha, 2008; Sayigh, 2007; Sayigh, 2013). Research on Palestinian displacement underscores how historical trauma extends into the present, manifesting through continuous processes of dispossession, systemic violence, and structural oppression (Hammami, 2005; Masalha, 2012; Sayigh, 2012; Shalhoub-Kevorkian, 2015, 2020; Giacaman, 2018). These include the destruction of homes, the establishment of military checkpoints, restrictions on freedom of movement, enforced closures, and the mass incarceration of Palestinians (Peteet, 2018; Abu Hatoum, 2021). The concept of an “ongoing Nakba” encapsulates this continuity, profoundly shaping Palestinian temporality, identity, and lived experiences in ways that challenge conventional understandings of historical trauma (Masalha, 2008; Sayigh, 2015; Barakat, 2021).

The persistent exposure to conflict and systematic violence has prompted scholars to critique traditional trauma frameworks that conceptualize trauma as a finite event. Specifically, conventional post-traumatic stress disorder (PTSD) paradigms fail to account for individuals subjected to daily trauma in contexts lacking safe spaces for recovery (Kimhi et al., 2010). In such environments, the framework of Continuous Traumatic Stress (CTS) offers a more nuanced lens. Developed by anti-apartheid mental health activists, CTS acknowledges trauma as an ongoing process encompassing both individual and collective dimensions—particularly in regions where colonial and militarized practices perpetuate constant states of threat (Eagle and Kaminer, 2013).

For Palestinians, the Nakba embodies a sustained source of continuous trauma deeply embedded in collective memory and identity. This ongoing trauma is perpetuated by Israeli policies that actively suppress Palestinian narratives while preventing the return of refugees and exacerbating their displacement (Qabaha, 2018; Hamdi, 2021). Such systematic practices ensure that the wounds inflicted during the Nakba remain open as Palestinians are repeatedly confronted with their dispossession. This ongoing displacement creates what Ghnadre-Naser and Somer (2016) identify as the intergenerational transmission of trauma, further shaping Palestinian consciousness and lived experiences. The current paper discusses childhood memories produced and shaped by this trauma of uprooting and loss. It demonstrates how these experiences continue to reverberate in contemporary Palestinian society. By examining these memories within the context of continuous trauma stemming from the Nakba, we gain deeper insights into how historical injustices influence present realities for Palestinians.

### Childhood, settler colonialism and the ongoing Nakba

Childhood in settler colonial contexts is profoundly shaped by systemic violence and dispossession. Settler states such as Australia, Canada, and the United States have historically targeted Indigenous children through policies designed to assert territorial control and impose social reordering (De Leeuw, 2009; Arvin et al., 2013). Colonial regimes strategically weaponized childhood—both as a metaphor and a direct target—to justify their claims of superiority and domination. By portraying Indigenous peoples as “primitive” and in need of paternalistic intervention, often under the guise of benevolent guardianship, colonial authorities legitimized forced separations, residential schools, and assimilation programs. These interventions disrupted the transmission of intergenerational knowledge and aimed to erase Indigenous cultures and identities (Liebel, 2021; Chen et al., 2017; Jacobs, 2009).

In Palestine, Shalhoub-Kevorkian (2019) contends that Palestinian children have consistently been subjected to the Israeli state’s structural and direct violence, beginning with the mass expulsion of Palestinians in 1948 and continuing through the ongoing Nakba. This violence is particularly acute in refugee camps, where children face curfews, night raids, movement restrictions, and daily humiliations, resulting in family fragmentation and deprivation of fundamental rights (Shalhoub-Kevorkian and Odej, 2018). Since October 2023, violence against Palestinian children has escalated dramatically in Gaza and the West Bank. By December 2024, 300 Palestinian children were detained by Israel. In early 2025, 27 had been killed in the West Bank. By March 2025, 119 children were in administrative detention under Israeli custody [DCI (Defence for Children International) Palestine, 2025]. According Palestinian Central Bureau of Statistics (2023) reports approximately 18,000 have been killed in Gaza, including infants and newborns, with many more injured or orphaned. Shalhoub-Kevorkian (2019, 2020) conceptualizes this ongoing process as “unchilding”—the authorized eviction of Palestinian children from childhood for political purposes, which amounts to the destruction of future generations as a means to eliminate colonized peoples. Since 1948, colonial strategies have aimed to enforce unchilding, forcing Palestinian children to face survival challenges far earlier than normal childhood would allow. Children who experienced the Nakba and those living in Gaza today share similar realities: witnessing mass death, enduring starvation and displacement, and suffering humiliation. Whether through the 1948 expulsions or ongoing siege and bombardment, Palestinian children exist in a world that denies them a protected childhood and compels them to develop complex ways of understanding and coping with colonial violence. This systematic violence is maintained by state and settler-colonial structures that racialize Palestinian children as inherently dangerous others, denying them their rights and humanity. As a result, Palestinian children become direct targets of state violence and political manipulation, stripped of their innocence and subjected to conditions that make their childhoods unlivable (Shalhoub-Kevorkian, 2019). This framework highlights how the political project seeks not only to control Palestinian bodies but also to erase their futures through sustained structural and physical violence.

### Childhood memories, trauma and resilience

Understanding how children navigate violence and adversity requires examining the intricate relationship between trauma, memory, and resilience. Childhood trauma fundamentally reshapes memory processes, often resulting in fragmented or nonverbal recollections that embody both suffering and the capacity for survival (Cohen et al., 2010; Van der Kolk, 2014). Unlike adult recollections, children’s memories are shaped by developmental stages, attachment dynamics, and the sociopolitical contexts in which they are formed (Schore, 2003). While some contemporary research attempts to move beyond pathologizing approaches, these frameworks remain rooted in Western epistemological assumptions that position Western knowledge as universal while rendering non-Western ways of understanding childhood trauma invisible or primitive (Burman, 2024). Recent research increasingly critiques Western trauma frameworks for universalizing childhood trauma experiences while marginalizing culturally specific understandings. Studies emphasize the evolving nature of traumatic memory (Weems, 2025). These findings support calls for new approaches to childhood trauma that recognize the political and cultural dimensions of children’s experiences rather than imposing universal developmental models. This growing body of critical scholarship demonstrates that trauma and resilience, therefore, are not opposites but interwoven processes—children actively engage memory to make sense of hardship, sustain identity, and seek continuity within their specific sociocultural contexts.

In colonial and conflict-affected settings, traumatic memory must also be understood as a political and collective experience. Fanon (1961) situates trauma within systems of racialized oppression, arguing that such memories serve not only as evidence of psychic injury but also as sources of resistance and identity formation. Survivors, as Stortz (2007) notes, carry both the memory of injustice and of survival—narratives that foster solidarity and intergenerational resilience. The concept of resilience itself has shifted from a fixed trait to an ecological process, emerging from dynamic interactions between children and their environments (Hertzig and Farber, 2013). Especially in protracted conditions of colonial violence and displacement, resilience entails more than coping—it reflects children’s capacity to endure and reframe suffering while maintaining ties to community, identity, and hope (Luthar et al., 2000).

Drawing on Fanon’s insights, it becomes clear that traumatic memory in colonial contexts carries the weight of collective history and political struggle. This recognition challenges simplistic binaries of pathology versus health and calls for a deeper appreciation of how children negotiate meaning, identity, and resilience within systems of oppression. To fully understand this complexity between traumatic memories and resilience among Palestinian children, this research centers their childhood voices and memories as primary sources of knowledge. Employing Burman’s (2017) ‘child as method,’ this study uses Palestinian childhood experiences as an analytical lens to examine how settler-colonial power operates through the systematic targeting of childhood, while revealing how adult Palestinian narrators deploy childhood memories to navigate ongoing displacement and resistance. By centering the childhood memories of Palestinian children, this research seeks to understand how children navigate the intersection of trauma and resilience within the ongoing context of displacement, offering insights into the dynamic processes through which memory becomes both a repository of pain and a foundation for continuity and hope.

## Materials and methods

This qualitative study employs a decolonial hermeneutic phenomenological approach to investigate the enduring impact of the Nakba through childhood memories of Palestinian survivors. Drawing on Umezurike’s (2021) framework, this methodology navigates the epistemological tension between European philosophical methods and decolonial critique by positioning both Heideggerian phenomenology and decolonial thought as resisting systems that privilege universal categories over lived particularities. Rather than adopting phenomenology as a philosophical doctrine, this study employs it instrumentally—appropriating its analytical contributions while “de-chaining from imperial designs” (Umezurike, 2021, p. 190). Heidegger’s concepts of Dasein (being-in-the-world), temporality, and the hermeneutic circle serve as interpretive entry points for accessing Palestinian lived experiences within settler-colonial contexts. This approach prioritizes Palestinian meaning-making and employs phenomenological interpretation to reveal childhood memories as existential responses to displacement and as sophisticated survival epistemologies that may challenge Western theoretical frameworks.

### Research design and participants

The research was conducted between 2019 and 2022, allowing for the careful development of trust with participants. The study involved 34 semi-structured, in-depth interviews with Palestinian refugees from Lydda who experienced the Nakba as children or were born in its aftermath. Lydda was chosen for its significance as a major site of 1948 displacement, enabling focused exploration of shared historical trauma within specific geographical and cultural contexts. Participants were recruited through purposive and snowball sampling via local community organizations and cultural centers in the West Bank. The sample included 18 first-generation refugees (10 women, 8 men, aged 73–88) who were 4–12 years old during the Nakba, and 16 s-generation refugees (9 women, 7 men, aged 49–72) born after displacement. All currently reside throughout the West Bank, mainly in refugee camps and surrounding communities. Data saturation was reached when no new existential themes emerged regarding childhood displacement and survival.

### Data collection and analysis

Interviews were conducted in Arabic in participants’ homes to create a familiar environment conducive to memory recall. Each interview lasted between one and three hours, with careful attention to participants’ emotional states and energy levels, especially among elderly first-generation refugees. Field notes documented non-verbal cues, emotional responses, and environmental factors that could influence interpretation. The interviews were then translated into English, presenting epistemological challenges. As Spivak (1993) observes, translation risks losing or distorting Palestinian experiential knowledge when English lacks concepts to fully capture key Arabic terms like Nakba (catastrophe), which hold profound cultural significance.

To mitigate this loss, key Arabic terms are retained alongside cultural explanations rather than substituted with inadequate English equivalents. Emotional and metaphorical expressions were carefully reviewed with bilingual Palestinian colleagues to preserve meaning. Nonetheless, some subtle nuances—particularly around collective identity and temporality—may still be diminished. Therefore, readers should consider quoted material as approximate representations rather than full access to Palestinian lived experiences.

Analysis followed a decolonial hermeneutic phenomenological approach prioritizing Palestinian meaning-making. The iterative interpretive process began with close readings of transcripts to access participants’ lifeworld experiences, advancing to deeper hermeneutic engagement with how childhood memories reveal core structures of being-in-the-world under settler-colonial conditions (Heidegger, 2011). Rather than imposing predetermined frameworks, Heideggerian concepts such as Dasein, temporality, and care served as interpretive lenses participants’ experiences could illuminate, challenge, or transform (Smith et al., 2021). This approach facilitated critical examination where Palestinian meanings might exceed Western theoretical expectations, with sensitizing concepts—such as unchilding (Shalhoub-Kevorkian, 2019), continuous traumatic stress (Eagle and Kaminer, 2013), and decolonial childhood studies (Burman, 2017)—dialoguing rather than dictating the analysis.

### Reflexivity

As a third-generation Palestinian researcher trained in Western phenomenological methods, I maintained ongoing reflexive awareness of how my positionality both facilitated cultural access and necessitated vigilance against imposing Western frameworks that risk colonizing Palestinian survival strategies. Reflexivity was practiced through regular debriefings with Palestinian colleagues and mental health and psychosocial practitioners working directly with Palestinian communities. These practitioners provided essential perspectives that helped ground interpretations in lived realities and community-held knowledge, supporting interpretations that honor participants’ meaning-making rather than serving Western theoretical validation.

This reflexive engagement revealed instances where Palestinian lived experiences fundamentally challenged my phenomenological assumptions, especially concerning temporality, childhood development, and trauma responses. Such moments required adapting analytical frameworks to better align with Palestinian epistemologies, rather than forcing experiences into pre-existing Western categories—exemplifying the practical enactment of a decolonial methodology.

### Trustworthiness and ethical considerations

Rigor was maintained through prolonged engagement with participants and their communities, the provision of rich, thick descriptions of existential contexts, reflective journaling, and the maintenance of detailed audit trails documenting analytical decisions. Member checking involved sharing preliminary interpretations with selected participants and community members to validate the processes of meaning-making rather than factual accuracy, thereby recognizing participants as co-creators of knowledge. Ethical approval for the study was granted by The Hebrew University Ethics Committee.

Consent procedures were culturally sensitive, respecting oral traditions alongside formal written documentation. Participants received comprehensive information about their rights, the study’s objectives, and potential risks and benefits, with particular attention to the sensitive nature of recalling childhood trauma. All interviews were conducted only after obtaining informed consent, and participants were reminded of their right to withdraw at any time without penalty.

To minimize potential harm and the risk of re-traumatization, participants were provided with information about accessible local support services. Given the limited availability of free psychological support in refugee camps and the broader West Bank region, additional precautions were implemented—especially for first-generation participants, many of whom are elderly and face significant barriers to accessing such services. The researcher, a social worker with specialized training in trauma and post-traumatic stress disorder (PTSD), conducted follow-up visits two to four days after each interview to monitor participants’ well-being and provide support as needed.

## Results

The childhood memories of the Nakba reveal experiences of unchilding, silence, and dehumanization that profoundly shaped survivors’ early years. Refugees recounted the trauma of being uprooted from their homes, the loss of belonging, and the disruption of family life. Many described childhoods marked by the loss of familiar surroundings, family, community, and place, and the daily challenges of survival in unfamiliar environments. Their narratives frequently highlighted the silence that enveloped their experiences, the pervasive sense of dehumanization, and the enduring effects of displacement on their understanding of home and family. The following findings illustrate how these themes emerged in the memories of both first- and second-generation refugees.

I will begin this section with the memories of Sarah, who was 7 years old during the Nakba and was uprooted from her home along with her sisters and grandmother. She was separated from her parents when her mother was forced to search the streets for her father, who had been missing for days, leaving the girls in the care of their grandmother. A few hours later, soldiers entered their home and compelled the family to leave. Sarah and her sisters remained separated from their parents for 4 months until their parents were finally able to find them. She recalls this moment of uprooting as the most vivid memory she has retained over the years—an image she has never forgotten. In her words, ‘*al-ḥuzn lā yunsā’* [*The sorrow is never forgotten*] an Arabic proverb that captures the enduring nature of grief, particularly the pain of loss and injustice. This expression reflects a sorrow that does not diminish with time but instead becomes a permanent part of a person’s consciousness. This section examines the childhood memories of Nakba survivors, exploring what they chose to remember, what they sought to forget, and how these memories continue to shape their lived experience and their psychological conditions.

### Loss of childhood: “I turned into an adult”

The childhood narratives reveal that refugees shared remarkably consistent memories of forced displacement from Lydda, characterized by abrupt departure on foot without possessions, typically under pressure and intimidation from Jewish soldiers. These collective memories document the abandonment of homes, communities, and loved ones in circumstances of extreme duress. The recollections of uprooting demonstrate how the childhood experiences of displaced children were fundamentally altered, representing a profound rupture that initiated an unfamiliar and traumatic developmental period. Participants’ memories consistently featured family separation, loss of loved ones, survival without adequate water and food, witnessing death, and the inability to continue their accustomed ways of life. These shared experiences mark the beginning of what can be understood as the systematic loss of childhood; a process through which children faced the trauma and brutality of war, and were forced to survive in such circumstances, where they turned into adults.

This theme explores how the Nakba abruptly ended childhoods, forcing premature maturation on young survivors. The analysis reveals two interconnected dimensions: first, the invisibility of children within systems focused solely on survival, where their physical and psychological needs were ignored; second, the systematic dehumanization that stripped children of dignity, humanity, and the protections typically afforded to childhood. These processes exemplify “unchilding,” whereby Palestinian children were transformed from protected dependents into premature adults focused on survival.

### The invisibilization of children

Layla was 7 years old during the Nakba. She was uprooted together with her parents, four sisters, and brother. They left Lydda in the middle of the day, under the scorching July sun, walking on foot to a neighboring village under the threat of soldiers. She began recounting her memories with the following words:

"The image of the gun pointed at my face is still clearly in my mind. When the soldiers entered our neighborhood to force us to leave, they were shouting and breaking and throwing people's belongings everywhere… When they entered our house, I was afraid and started to cry loudly. One soldier put his gun to my face and shouted at me, saying words that I didn't understand… I remember how I froze and couldn't move, and my father was forced to grab me and carry me as we fled from the neighborhood… The soldiers continued to kick children and shout—they didn't care that we were just children. It was as if we didn't exist, our tears, our screaming…all of it was unseen to them. We were treated as if we were nothing more than barriers to be removed."

Layla’s memory exemplifies the invisibilization of the child as a systematic structural process targeting children. Building on existing literature on invisibilization in colonial contexts (Heiss and Herzog, 2021), I apply this concept specifically to Palestinian children, denoting the denial of their existence and silence their voices. This process is manifested in Layla’s memory and others’, through the systemic suppression of children’s physical and psychological needs and the structural marginalization of their status as children. Ahmad, 10 years old at the time, similarly recalls:

"I tried to take a small piece of bread and put the bread in my clothes, but the soldiers who prevented us from taking anything from home saw me and took the bread and shouted in my face rūḥ [go in Arabic]."

Ahmad’s account underscores how his basic needs and humanity were disregarded, reinforcing his sense of invisibility. The theme of invisibilization articulated by Dalal, who was 9 years old during the Nakba. She recalled experiencing herself as a “khayāl” [shadow] in the midst of the violence surrounding her family’s displacement. While fleeing with her parents and two sisters, she was shot in the right hand by soldiers firing at civilians at the entrance of their neighborhood. Reflecting on that moment, she recounted:

"When I felt the bullet and saw the blood on my hand, I was terrified and screamed. My father, who was trying to protect us from the shooting, put his hand over my mouth and forced me to remain silent throughout the journey to Ramallah. I was bleeding; no one saw me, no one tried to help. My parents and family were focused on survival. I still feel the pain in my hand even now, but I was like a shadow — unseen."

Dalal’s narrative illustrates how chaos, trauma, and the imperative of survival rendered children’s suffering invisible, contributing to a profound sense of erasure both physically and emotionally.

Sabri’s recollection adds a poignant dimension to the collective memories of forced displacement and loss. Born during the Nakba and raised in a worn-out tent between two Palestinian villages, Sabri reflects on the erasure of childhood itself: “*I do not know what childhood means… We were born into suffering. They want us to disappear since we left Lydda.*” His words underscore the profound rupture in the experience of childhood caused by displacement and protracted trauma. Like Layla, Ahmad, and Dalal, Sabri’s narrative illustrates how Palestinian children were not only physically uprooted but also socially and psychologically marginalized, rendered invisible within a context of ongoing violence.

This invisibilization contributed to the systematic erasure of children’s suffering, agency, and voices, deliberately silencing their experiences. Together, these memories reveal the process of unchilding, whereby Palestinian children were forcibly transformed from protected dependents into premature adults, burdened with the trauma and responsibilities of survival in a world marked by loss, violence, and dispossession.

### The dehumanization of children

The memories reveal how the extreme conditions of displacement systematically dehumanized Palestinian children, reducing them to a state beneath human dignity through the imposition of inhuman and degrading circumstances. These conditions forced children into survival situations so dire that some refugees described them as resembling animal-like existence. This dehumanization, as articulated by Haslam (2006), entails the denial of uniquely human attributes, coercing children into experiences that strip away their humanity and erode their agency, identity, and moral worth. Said, fourteen years old during the Nakba, recalled harrowing memories of thirst, hunger, and shame. He described how, amid the brutal conditions of displacement, parents were forced to resort to inhumane measures to help their children survive:

"It was July… we walked for days to reach the Ramallah region, without water, food, or any sustenance that could help us survive. I remember losing consciousness twice from thirst. My father, like others, found a way to hydrate us. He asked everyone, including my mother, to urinate into something that looked like a cup he found on the road, and we drank our urine during the march."

Said mentioned that these memories from the Nakba period remain deeply ingrained in his consciousness, accompanied by a profound sense of shame and pain that continues to affect him. Similarly, Amneh, 9 years old at the time, recalled how survival often depended on unimaginable sacrifices and inhumane coping strategies, which inflicted feelings of inferiority and helplessness on children—psychological effects that the systematic process of unchilding sought to produce. She said:

"We didn't eat for the whole day. We were very young; I was the oldest child in my family… My brothers didn't stop crying because of hunger, many children were crying. My father and two other men found a dead goat on the road… They were afraid of the soldiers, so they didn't make a fire to prepare the meat… We ate the raw meat… only animals do that… Since that day, I don't eat any type of meat. I still feel the blood in my mouth."

Alyah, 6 years old at the time, recalled a harrowing experience during her family’s forced uprooting from Lydda. Along with her mother and five siblings, they were compelled to leave their home without any belongings, including water. After hours of walking, the children were desperately thirsty. Their mother searched for water and eventually found a pothole filled with unsanitary water. Knowing the risks, she soaked her clothes in the contaminated water and returned to her children, making them suck the water from her wet clothes to hydrate. Alyah recounted:

"When people saw my mother’s wet clothes, they attacked her and started sucking the water from her clothes. My poor mother tried to escape and screamed. We were terrified and crying. She took off her Thobe [traditional dress] and threw it at the people. I still remember well how people were crawling to get my mother’s Thobe like ghosts. We thought they would kill my mother. The poor people turned into vicious animals in order to survive."

Safaa, who was born 10 years after the Nakba, describes how her parents’ memories of dehumanization have become part of her own memory and ongoing psychological experience, one that did not end with the conclusion of the war. In her words:

"I was born in the camp. We were nine people in one bedroom, living in boxes. I didn’t go to school because my father couldn’t afford shoes for all the children, so I had to stay barefoot for a long time. It was a cruel circumstance. I remember standing in the UNRWA line to receive milk or food. I have never seen the sea; I have never left the camp. They [the Israelis] decide when we can move, what we can eat, and where we should live. Even now, young soldiers, just 18 years old, catch you at the checkpoint and play with you like a toy. We are no different from animals. It’s the same situation since the Nakba."

This narrative illustrates how policies of restriction and control perpetuate the structural dehumanization of Palestinian children across generations, creating ongoing inhuman conditions that many refugees describe as resembling animal-like existence. The memories of Palestinian children during the Nakba reveal a systematic process of “unchilding”; the complete erasure of childhood through intertwined mechanisms of invisibilization and dehumanization. Children’s suffering went unseen, their voices unheard, and their basic needs ignored. Simultaneously, children endured conditions so degrading that survivors used animal metaphors to describe their existence, drinking urine and eating raw meat. This transformation from protected dependents into prematurely burdened adults is captured by George, an eight-year-old boy left behind in Lydda, who said, “*Since the Nakba I changed, I felt that I must grow up and be an adult*.” His words underscore how the Nakba did not merely interrupt childhood but systematically erased it, compelling Palestinian children to abandon their innocence and assume adult responsibilities in a world that denied them the recognition and protection essential to childhood.

### Silencing childhood: “to be silent is to survive”

children memories reveal that silencing became deeply embedded in every aspect of their displacement, operating through multiple mechanisms, including systematic intimidation—a psychological tactic employed by Jewish militias to control movement and suppress voices, targeting both immediate reactions and long-term abilities to process ongoing trauma. Their narratives demonstrate that silence was not merely the absence of sound or speech, but an active force that shaped survival strategies and psychological experiences throughout the ordeal. Some refugees recalled being told not to cry or speak about their experiences as children, describing “crying in silence” and feeling compelled to suppress their emotions. Others noted that they were unable to express pain or seek help, describing a persistent sense that their childhood suffering was silenced and unacknowledged. From a psychological perspective, silence emerges in these memories as both a defense mechanism against the trauma of loss, shock, and collective suffering, and as a survival strategy necessary to endure the horrors of the Nakba, the dehumanization they faced, and the deep psychological wounds it caused.

For this theme, the discussion will focus on how silence and silencing functioned as survival strategies from the refugees’ perspectives, illustrating the complex ways in which imposed silence shaped their experiences and coping mechanisms during and after the Nakba. Refugees described survival strategies centered on the suppression and silencing of their senses—such as controlling their eyes and inhibiting their sense of smell—to avoid detection and protect themselves amid violence and displacement. Additionally, silence extended to the suppression of memories, where painful recollections of suffering and trauma were deliberately muted or left unspoken as a means to manage overwhelming grief and facilitate endurance. These dual forms of silence—both sensory suppression and memory silencing—were crucial for psychological survival in the face of dehumanization and ongoing trauma.

### Silencing the senses

During the Nakba, Ali and his family walked for 2 days to reach Ramallah. The journey on foot from Lydda to Ramallah, especially with children, was arduous and nearly impossible. The family lost their way twice, forcing Ali’s father to change their route to ensure they arrived safely. Ali, who was 8 years old at the time, recalled the road between the Palestinian villages with these words:

"The road was full of bodies—I couldn't count them. It was a horrible scene you can't imagine; most were children and women."

Ali could not bear the pain of seeing the dead, especially infants and children, he silenced his senses to cope with the horror and to repress the fear and guilt he felt:

"I closed my eyes every time I saw a dead body. I pretended not to hear the screams of the people. I thought it would be better than seeing others dying and not helping them, and feeling ashamed of myself. The smell of dead bodies accompanied us all the way. It was very hot, and the bodies began to decompose quickly. Despite all my efforts to forget, I still remember the smell clearly".

Ali’s deliberate avoidance of seeing, hearing, and smelling illustrates how silencing the senses became essential for survival and enabled children to function in circumstances that would otherwise paralyze them with fear.

Similarly, Susan’s experience reveals another dimension of sensory silencing. She was 6 years old during the Nakba when her foot was injured after walking barefoot for hours. Her father was forced to carry her for much of the journey. After hours of walking, her father, became tired and thirsty and asked the family to rest under a tree. Susan, in pain and exhausted, kept crying. A refugee walking with them approached her father and said, *“Leave the girls under the tree and keep the boys; people are abandoning their children, but God will protect them until we come back.”* Susan heard this and was terrified. She shared her memories and feelings:

"I was afraid. I pretended not to hear the man, but all the way I kept thinking, 'What if he leaves me like the others?' My foot was throbbing with pain, but I shut my ears and my mouth. I didn't say a word, stopped crying, and continued in silence. I was afraid my father would get tired and leave me."

As she spoke, Susan cried and showed me her injured leg, which worsened over the years due to lack of proper medical care and complications from diabetes. Although her father did not heed the man’s suggestion and carried on with her, the fear and anxiety continued to dominate Susan’s experience. She blocked out the sounds and scenes around her and did not communicate with those nearby:

"The images of abandoned children were always in my head. I couldn't look anymore… I was terrified, thinking about that man and my father. I closed my eyes until we reached Ni'lin [Palestinian village], and I didn't cry."

Following this exchange, Susan stopped crying or speaking altogether. She ignored her pain and the suffering around her as a way to survive and avoid being abandoned like the others. George’s memories demonstrate the most extreme form of silencing the senses. At the age of eight during the uprooting, he witnessed the death of his mother from thirst and the killing of his uncle by soldiers. Forced to abandon their bodies without a proper burial or the opportunity to bid them farewell, his reluctance to share detailed accounts of these traumatic events, and how silencing his eyes and ears helped him to continue his journey with the family, in his words:

"When my father realized that my mother Tarez had passed away… he put her body under a tree… we left her… even without a cover, my father didn't allow us to pray next to her. My sisters and I were crying loudly over our mother's body… but my father screamed and told us they [the Jews] will come after us and kill everybody… I saw them killing my uncle that same day, I realized it was the truth. I shut my mouth and cried in silence all the way to Ramallah… when we passed dead bodies or injured people I closed my eyes, I didn't want to see or hear people who were dying. I was moving like a machine without hearing or seeing anything."

George described how silencing his senses turned him into “a machine without hearing or seeing anything” after witnessing his mother’s death from thirst and his uncle’s murder in a single day. His father’s command to suppress mourning forced George to emotionally disconnect as a means of survival. This total silencing of senses and feelings illustrates the profound trauma of childhood—the suspension of a child’s emotional self to preserve physical life.

The memories of Ali, Susan, and George reveal how silencing the senses became an essential survival strategy for Palestinian children during the Nakba, allowing them to endure circumstances that would otherwise overwhelm their psychological capacity to function. This sensory silencing operated across multiple dimensions—visual, auditory, and olfactory—and ranged from selective avoidance to complete sensory shutdown. The children’s accounts demonstrate that this was not passive numbing, but active psychological work that required tremendous effort and came at significant cost. While this mechanism enabled immediate survival during the crisis of displacement, it also established patterns of suppression that would extend far beyond the journey from Lydda, creating the foundation for the systematic silencing of memories and experiences that would characterize the refugees’ post-Nakba existence. The silencing of the senses during displacement thus represents the first layer of a broader politics of silencing that would continue to shape Palestinian childhood for generations to come.

### Silencing the memories

While the silencing of the senses enabled children to survive the immediate trauma of displacement, a different form of silencing emerged in the aftermath of the Nakba—the systematic suppression of traumatic memories and experiences. For both first and second-generation refugees, silence became a dual survival strategy: internally, it provided psychological protection from overwhelming memories of loss, death, and dehumanization; externally, it offered protection from ongoing Israeli threats and surveillance that made the open expression of Palestinian experiences dangerous.

Layla recalled how after resettling in Jalazone Camp, the trauma of loss, the pain, and the deep sense of helplessness were overwhelming. She described these feelings as ‘a fire [Nār in Arabic]’ that never stops burning—a relentless flame that scorches through every moment, leaving behind ashes of fear, loss, and disorientation. She explained:

"She recalled how she preferred not to remember anything related to the uprooting and their journey to the camp: "After what happened to us, I couldn't even remember what happened. I didn't talk about what I experienced or saw… I saw many horrible things. What can be more painful than seeing my cousin's body in the street? She was my friend… I couldn't handle these memories, I forced myself to forget… I remember that I never talked about this in front of my parents… I pretended that I don't remember… and I grew up and never talked about what I remember".

Layla’s deliberate forgetting represents a protective silencing strategy where suppressing traumatic memories became essential for psychological survival. By pretending not to remember and refusing to speak about her experiences, she created a barrier between herself and the overwhelming pain of reliving the trauma. This conscious act of silencing allowed her to function in daily life while avoiding the emotional devastation that would come from repeatedly confronting memories of loss, death, and helplessness.

The same experience of silencing the memories was described by Majed, who was fourteen years old during the Nakba. During the interview, Majed kept asking me:

"Why do we need to remember? What will happen if I talk about what happened? I don't want to remember, I just want to be relieved… since the war I saw people here talking about the Nakba, and they became sadder and depressed. Nothing happened—they didn't return to their homes and no one cared about their suffering. I don't want to be like them."

Majed’s decision to remain silent about his trauma reflects a deliberate survival strategy, shaped by his belief that remembering only intensified suffering without providing relief or justice. His refusal to engage with painful memories was not passive avoidance but a pragmatic effort to protect his psychological well-being in a context where remembrance offered no healing. This strategy of protective silencing extended to the second generation, as demonstrated by Sanaa, who was born 2 years after the Nakba in al-Amari refugee camp. During the interview she recalled very cruel living circumstances: the lack of food and shelter, the separation of the family when her father and two brothers were forced to leave for months in order to work and bring urgent necessities, and the persistent fear and hope of returning to Lydda. Despite not witnessing the uprooting, she described silencing the memories with the following words:

"My grandmother all the time was telling us about *alblad* [Lydda] and how they forced them to leave, and how her neighbor forgot her child at home from fear and they couldn't go back to bring him. As a child I was panicked, it was very fearful to hear these memories… we also suffered in the camp, our childhood was very hard. I decided to forget my grandmother's memories and the camp memories… I didn't talk to my children about it, I don't want them to have these memories. It's very hard now after all these years to remember how I was barefoot and hungry, how I wanted to go to school but I couldn't."

Sanaa’s experience shows how silencing memories extends beyond the first generation, encompassing inherited trauma. Her choice to withhold these stories protects both herself and her children from reliving the pain, enabling survival amid ongoing hardship.

While Sanaa’s silencing was primarily driven by personal protection and family preservation, Emad’s (born during the Nakba) approach to memory suppression revealed another dimension of survival—protection from external political threats. Emad described silencing the memories as a survival strategy necessitated by the politics of the Israeli authorities that threatened refugees’ lives since the Nakba. He mentioned how memories, especially about the past, the war, the uprooting, and the horrors of the Nakba, became taboo subjects that could endanger those who spoke about them. Reflecting on this, Emad said:

"I remember all my grandfather's stories and my father's stories about Lydda, and how they left their homes… but here we can't talk… the walls have ears… I learned it from people who talked and paid for it…. some were arrested, some were forced to leave their work and never found another… if you want to live you should not remember your family's past, your origins. It's like Lydda wasn't our homeland… we weren't there; we didn't live there…"

Emad’s words reveals how silencing memories became a necessary survival strategy that required denying not only traumatic experiences but also Palestinian identity and connection to their homeland. The consequences he witnessed—arrests and job losses of those who spoke about their experiences—reinforced his understanding that silence was essential for survival. For Emad, suppressing memories and denying his family’s past became the price of being able to continue his life without facing persecution, demonstrating how political threats transformed silence from a coping mechanism into a daily survival.

The testimonies of Layla, Majed, Sanaa, and Emad reveal how silencing memories became a vital survival strategy across generations. For first-generation survivors, silence offered protection from the psychological weight of trauma, while second-generation refugees suppressed both inherited and personal memories to shield themselves and their children. Emad’s story highlights how political threats turned silence into a necessity, where remembering could lead to persecution. These narratives show how memory suppression became a lasting feature of Palestinian refugee life—a means of coping with trauma and resisting ongoing oppression.

### The child that remained: memories countering trauma

"If, one day, the people will to live, then fate must obey… Darkness must dissipate, and the shackles must break… I love this poet — his words speak to our reality. We were so young when the Nakba happened, but we are still here. We never gave up…We still remember who we were and where we came from, we were born and raised in Lydda, our childhood was in Lydda…"

These are the words of Sarah, who was 7 years old during the Nakba and whose words I began this section with: “*the sorrow never forgets*.” Like other refugees, Sarah as a child witnessed both the Nakba and its aftermath. Her descriptions of the Nakba’s memories reveal that memory in her consciousness works as two sides of the same coin: painful traumatic memories, and a site of power and resistance against erasure and unchilding. Her insistence that “we are still here” and “we never gave up” transforms childhood memory from a site of loss into a declaration of survival and continuity.

Refugees’ childhood memories position remembering and past experiences as a dynamic space where silencing and suppression coexist with acts of resistance and resilience. Despite trauma, displacement, and ongoing oppression, refugees preserved fragments of childhood—not the lost childhood of the Nakba itself, but the childhood they chose to remember: the child who remained connected to their homeland. These preserved memories function as acts of defiance against the continuous political erasure of Palestinian identity and the distortion of childhood experiences.

The refugees’ narratives demonstrate how childhood memories of their homeland, Lydda, serve as protective psychological spaces where their pre-trauma selves remain intact and continue to support resilience over time. These memory sanctuaries preserve not only the factual details of early life but, more importantly, the emotional landscapes of safety, joy, and belonging that existed before displacement and ongoing oppression. Naela, who was 10 years old during the Nakba, lived in her grandparents’ house with her extended family. She described how “the big house included all the family’s children,” and how she spent her days playing with them in the garden. Recalling her happiness, she said:

"I lived ten years in Lydda… I had the most beautiful childhood. We were a big family, living together and spending time together… I remember our grandmother in the garden telling us stories. My grandfather brought a teacher to our home to teach us reading and writing. Those were good days—worth remembering."

Naela described the uprooting as the shock of her life, saying that their world was destroyed and nothing ever returned to the way it was—especially after the separation of her family. Yet despite the pain, her childhood memories continued to strengthen her in the aftermath of the Nakba:

"I passed through many cruel circumstances… You can say that all my life has been a sense of loss—losing al-blad, my family, my husband, and my son. Since the Nakba, it has been like this. It wasn't easy to live here [in the camp]; it's not our home… Remembering Lydda gives me relief and makes me feel safe; it brings back that happiness. When I feel overwhelmed by what is happening to us in the camp, I close my eyes and imagine I'm sitting in our garden, then I calm down."

The embodied nature of these memories extends beyond psychological recollection into physical practice. Naela mentioned that she continued styling her hair the same way her grandmother had done for her in Lydda, maintaining this practice many years after the Nakba and into adulthood. She described this ritual as a way of preserving memories and remaining connected to her childhood self.

The concept of memories serving as a psychological sanctuary illustrated through Ali’s recollections. He describes Lydda as ‘paradise’ where everything was available. He went to school, played with children for long hours in the neighborhood, and participated in ceremonies. He described how the community was very collective and supportive of each other, which made his childhood experience special: “*I felt the freedom…as a child, I was free to play and move. Lydda was a safe place; everybody cared for children*.” He explained how these memories of paradise and safety transformed into a safe psychological space:

"The memories are important for us…they help us to feel comfortable and belong to our community and city. The belonging makes me feel safe in this cage that we live in today. We need to keep passing these memories to our children and offspring—it helps them to feel the belonging."

Like Naela, Ali’s connection to memory manifests through embodied practices. He continued playing his favorite childhood game from Lydda called ‘the seven stones’ and taught it to his children. For him, this childhood game represented an important component of his memories, serving as a tangible link to his past that he could physically recreate and share with the next generation. Majed reinforced these themes during his interview, emphasizing how memories counter the psychological effects of dehumanization. He explained that while the trauma they suffered during and after the Nakba left them psychologically overwhelmed, remembering their origins and childhood in Lydda restored their sense of belonging and dignity:

"Since 1948 we are refugees…they made us homeless…but we know well that's not true. We have origins, we have homes in Lydda. We were there and spent our childhood in the city. I remind myself and my children all the time that we have roots, we are from Lydda. This makes me feel strong, makes me feel human."

Majed also described how actively sharing these memories strengthened their preservation. He explained that telling stories about the past and recounting Palestinian literature he knew as a child to others—people in the camp, his friends, and children—helped him continuously recall and maintain these memories. This narrative sharing transforms individual memory into collective practice, creating community bonds while ensuring the memories remain vivid and accessible.

Narratives of second-generation refugees indicate that inherited memories from the first generation play a crucial psychological role in their everyday lives. These memories provide refugees with a sense of belonging to a place and people, fostering feelings of humanity and continuity in lives characterized by exile and ongoing violence under occupation. Suha, 64 years old at the time of the interview, never visited or lived in Lydda, but she grew up listening to her father’s stories and memories about the past. Suha preserves those memories as if they were her own, indicating that these inherited narratives provide her with a picture of the normal life she never experienced and serve to humanize her experience as a refugee:

"I remind myself and my children of my parents' stories about our house in Lydda. It makes me feel that I have something worth living for and not to give up on this messy and crazy life here in the camp. It makes me feel relief."

The transmission of memory extends beyond storytelling to embodied practices. Suha’s father taught her to make dolls from simple fabric—the same type of dolls that her grandmother had made for him in Lydda. Suha described these dolls as her inheritance from her father’s memories, explaining that creating them made her feel belonging and provided emotional relief. Through this tactile practice, second-generation refugees transform inherited memory into embodied knowledge, creating psychological safe spaces that transcend physical displacement.

The narratives show that both first- and second-generation refugees continue to draw upon memories of pre-1948 Lydda as essential psychological resources in their daily lives. Participants reported that these memories provide emotional support, offering comfort, stability, and a sense of inner safety amid ongoing adversity. By mentally returning to moments of happiness, security, and belonging from their early lives, refugees are able to regulate distress, manage the psychological impact of trauma, and sustain hope across generations of displacement. This reliance on positive childhood memories functions as a coping mechanism, helping individuals preserve psychological well-being despite persistent instability and loss.

## Discussion

### Childhood as counter-method: Palestinian memory challenges Western knowledge systems

This study reveals that the Nakba marked a pivotal turning point in the childhoods of Palestinian refugees. The memories documented in the narratives illustrate systematic processes of *unchilding* (Shalhoub-Kevorkian, 2019), manifested through the loss of childhood, premature assumption of adult responsibilities, dehumanization, and invisibilization. Analysis of these narratives demonstrates that childhood memories serve as complex sites where experiences of displacement-related trauma intersect with adaptive survival mechanisms, including selective sensory silencing and strategic memory suppression, that enabled children to navigate impossible circumstances. Importantly, these strategies not only facilitated survival but also helped to build resilience by preserving essential elements of identity and community connection, sustaining refugees across generations of ongoing settler-colonial violence.

These findings challenge conventional Western trauma frameworks, which conceptualize traumatic experiences as discrete, time-bounded events amenable to therapeutic resolution (Giacaman, 2018). This limitation is compounded by cross-cultural approaches that often frame the psychological experiences of people in “developing countries” through rigid distinctions between the “East” and the “West,” uncritically imposing psychoanalytic concepts rather than engaging with people’s subjective experiences (Mihalits, 2017). Palestinian children’s memories instead exemplify what Fanon (1961, p. 4) identified as the structural nature of colonial trauma—violence that is not episodic, but constitutive of the colonial relationship itself. Fanon described this as “sociogenic” trauma, meaning trauma that arises from oppressive social structures rather than individual pathology. For example, when Ali states, “since the Nakba, I could not remember anything beyond the loss of our homes and land,” when Layla pretends not to remember the Nakba, or when Majed expresses ambivalence and hesitation toward remembering, they are not articulating psychological dysfunction. Rather, they embody what Fanon recognized as the colonized subject’s disrupted relationship to time, memory, and selfhood under ongoing colonial violence.

This section discusses how Palestinian childhood memories reveal the inadequacy of conventional Western psychological frameworks for understanding children’s experiences under ongoing colonial violence. It argues that Western frameworks not only misinterpret Palestinian children’s experiences but actively obscure the sophisticated survival epistemologies they develop under impossible conditions.

As a social worker educated within a Western system and trained in welfare practices grounded in Western knowledge, research, and theories, I was accustomed to clearly defined rules regarding child abuse, childhood trauma, and appropriate interventions for children and families. While these frameworks often strive to be “culturally and religiously sensitive,” analyzing the narratives of refugees proved to be a complex and challenging task that requires more critical and nuanced perspectives to fully understand the cultural, historical, and political dimensions involved. For example, when I first heard George’s memory about his mother’s death and how his father prevented him from saying goodbye, my initial reaction was to feel sorry for this seemingly powerless child experiencing traumatic loss. I interpreted his silent response as a defensive mechanism and judged the father as cruel, someone who could have acted with greater sensitivity toward the child’s shock. However, after our second meeting and upon deeper reflection on his description of their circumstances, I began to see the child within George differently—recognizing the complex survival strategies embedded in this memory and the remarkable ability of this child to continue his life. Rather than pathology, I saw how George was building a safe psychological space by recalling positive childhood memories with his mother.

This shift in understanding raises critical questions about the production and validation of knowledge in contexts of ongoing colonial violence: Who determines what constitutes legitimate knowledge about childhood trauma and survival? How do Western academic and professional frameworks silence or pathologize the survival strategies that enabled Palestinian children to endure impossible circumstances?

Drawing on Michel Foucault’s analysis of power/knowledge relations in *Discipline and Punish* (1977), these Palestinian childhood memories reveal how Western professional frameworks—including social work, psychology, and trauma studies—function as disciplinary mechanisms that normalize Western ways of knowing while rendering alternative knowledge systems invisible or pathological. Edward Said’s critique of colonial discourse similarly highlights how Western knowledge production dominates and marginalizes the colonized “Other.” This disciplinary power operates not only through direct repression but through the establishment of what constitutes ‘legitimate’ knowledge about childhood trauma and appropriate responses to it. The study shows how Palestinian children developed practices such as sensory silencing and strategic memory suppression in response to their psychological situation. These were not merely unconscious pathological or primitive defense mechanisms, as Freud (Di Giuseppe et al., 2021) and other theorists have suggested, but rather conscious sophisticated survival strategies adapted to conditions of ongoing violence and dispossession. Contemporary applications of Freud’s theorizing of the “primitive” construct it as the container for all that is not civilized, signifying qualities or traits such as irrationality, hypersensitivity, and psychological, intellectual, or social inferiority (Tummala-Narra, 2022). This approach maintains and even strengthens settler-colonial psychological policies toward Palestinian refugees.

The conscious act of recalling these memories by refugees, and their articulation of silencing as a deliberate strategy to survive, live, and continue, reveals forms of knowledge that have been systematically excluded from Western frameworks. This study suggests understanding the children’s responses as recalled by the refugees not as pathological responses requiring therapeutic intervention, but as knowledge that foregrounds resilience and agency. It challenges Western frameworks that pathologize these strategies as dissociation or avoidance, calling instead for careful consideration of cultural and contextual differences. This invites a critical re-examination of trauma and intervention approaches in settler-colonial contexts, emphasizing the importance of socio-political realities and community-based coping mechanisms.

Rethinking childhood memories of acting against dehumanization and invisibility in order to survive reveals how Palestinian children were not passive victims but active agents, developing acute awareness and consciousness of the colonial forces shaping their reality. These experiences directly counter universalist frameworks that construct what called modern notion of childhood as a state of victimhood, innocence, passivity, and powerlessness (Greer, 2007). As Burman (2024) argues, the figure of the child is deeply implicated in the production and maintenance of social, political, and racial hierarchies, with dominant Eurocentric models universalizing culturally specific notions of innocence and dependency. By foregrounding the agency, resilience, and political consciousness demonstrated in Palestinian childhood experiences, we unsettle these naturalized assumptions and create space for alternative, decolonial understandings that reveal how childhood functions as a site where colonial power operates and where sophisticated survival strategies emerge under impossible conditions. Palestinian children’s acts of resistance—whether through bodily presence, strategic silence, or daily navigation of violence—demonstrate lived resistance and the refusal to be erased or rendered invisible by settler-colonial forces.

When nine-year-old Dalal was injured by a bullet in her hand during displacement, she described herself as becoming “a shadow”—not an injured child requiring medical and psychological protection and intervention, but an invisible presence whose suffering went unrecognized even by her own family, who were focused on survival. Significantly, Dalal never blamed her father for putting his hand over her mouth to silence her cries of pain, demonstrating that even as a child, she understood the survival imperative governing their circumstances. Her ability to recall this moment as an adult without resentment reveals a form of border consciousness that grasped both her need for care and the impossibility of receiving it under colonial violence. This transformation from “injured child” to “shadow” and the articulation of such harrowing survival practices exemplify what Mignolo (2012) identifies as border thinking—knowledge that emerges from existing between the categories colonial systems recognize and the lived realities of those systems’ violence.

Dalal’s experience reveals the colonial difference at work: Western child protection frameworks would classify her as a trauma victim requiring immediate intervention and might pathologize her father’s actions as harmful rather than protective, while Dalal’s consciousness allowed her to understand and give meaning to survival logic that made care impossible under settler-colonial assault. Dalal’s childhood understanding that her father’s silencing was necessary for survival represents a sophisticated border epistemology that Western child protection frameworks cannot accommodate (Mignolo, 2012; Abebe et al., 2022).

Other childhood memories, such as drinking urine or eating raw meat with blood, vividly reveal Palestinian children’s understanding and awareness of the unchilding processes they experienced, demonstrating how settler-colonial violence forced them into survival situations that contradicted Western notions of childhood innocence and passivity as a state requiring external rescue and protection. War and settler-colonial violence severed their childhood and transformed them into adults, forcing them to act with adult-like responsibility to survive. Ali recalled his duty to bring water for his sisters, while Susan described how she prevented herself from crying and pretended to handle pain like adults do, all as deliberate strategies to survive. These examples call into question Western perspectives on childhood protection, which fail to account for such adaptive agency in contexts of ongoing violence (Shalhoub-Kevorkian, 2019).

The consciousness demonstrated by Palestinian children ultimately constitutes a refusal to accept Western analytical frameworks as universal approaches for understanding human experience. To understand how this can be understood as refusal, I draw on Audra Simpson’s (2014) analysis of Indigenous refusal, which demonstrates that such refusal is not merely rejection but a deliberate, political, and ethical stance that protects sovereignty and self-determination by resisting the imposition of colonial epistemologies. The memories examined in this study exemplify such refusal through what can be termed countering trauma, a process whereby both first and second-generation Palestinian refugees refuse to accept erasure, elimination, or psychological destruction. Despite experiencing profound trauma, they refuse to relinquish memories of freedom, “good days,” and descriptions of Lydda as “paradise.” Rather than interpreting these recollections as idealization, denial, fantasy, or trauma-induced distortion, Simpson’s framework reveals how preserving positive memories constitutes a form of refusal that protects what is sacred from colonial conquest.

This aligns closely with what decolonial theorists such as Mignolo (2012) call “epistemic disobedience.” Epistemic disobedience refers to the conscious rejection of colonial ways of knowing and the assertion of knowledge systems that emerge from the colonial difference itself, rather than from within Western epistemological frameworks. Mignolo argues that “delinking” from Western categories of thought is necessary to escape the coloniality of power, and that epistemic disobedience opens space for new beginnings rooted in Indigenous and other marginalized epistemologies. In the Palestinian children’s case, as demonstrated in this study and others (Shalhoub-Kevorkian, 2019), the Nakba initiated a continuous systematic process that affects every aspect of children’s lives and psyche. Perhaps the most observable contemporary example is the ongoing war on Gaza and the unimaginable suffering of Palestinian children. This specific political context demands new knowledge based on what Paulo Freire’s pedagogy calls for: education rooted in the lived experiences and agency of the oppressed—Palestinian children—rather than imposed from above through colonial or authoritarian frameworks.

Building on Mignolo and Freire, Burman (2017)'s decolonial analysis of childhood demonstrates how this epistemic disobedience extends specifically to challenging Western constructions of childhood. Palestinian children’s survival strategies constitute what Burman calls “childhood counter-narratives” that refuse Western developmental paradigms and assert alternative understandings of children’s capacities, agency, and knowledge under conditions of colonial violence. This decolonial childhood perspective reveals how Palestinian children’s experiences challenge not only Western trauma frameworks but the entire colonial edifice of childhood studies that positions Western developmental models as universal while pathologizing non-Western ways of understanding children’s lives and capacities.

However, in this analysis I do not suggest a wholesale rejection of Western theories or therapeutic interventions. Rather, I call for researchers and professionals to approach these frameworks with critical awareness of their limitations and cultural specificity. The goal is not to abandon Western knowledge entirely, but to recognize it as one among many ways of knowing, while actively incorporating Indigenous and local epistemologies into practice. In other words, this approach aims to humanize Palestinian children’s narratives by understanding and seeing the child as a whole person with all of the intertwined complexity of intrapsychic and interpersonal dimensions (Tummala-Narra, 2021). This requires what Linda Tuhiwai Smith (2021) calls “methodological reflexivity”—constant critical self-awareness by researchers and practitioners of how Western frameworks may silence or pathologize non-Western ways of understanding childhood, trauma, and healing. For Palestinian children specifically, this means integrating Palestinian cultural understandings of childhood, family, community healing, and resistance alongside carefully selected Western approaches that can support rather than undermine Palestinian ways of knowing. The challenge lies in creating what Bhabha (2004) calls “hybrid” approaches that honor both Palestinian epistemologies and useful Western insights without allowing the latter to dominate or erase the former. This demands ongoing dialogue with Palestinian communities, and specifically with children as Burman suggests, about what forms of knowledge and intervention truly serve their needs and self-determination.

## Conclusion

This article demonstrates how Palestinian refugee childhood memories challenge Western knowledge systems while revealing sophisticated survival strategies developed under ongoing settler-colonial violence. Palestinian children’s experiences of “unchilding”—invisibilization, dehumanization, and forced premature maturation—cannot be adequately understood through conventional Western trauma frameworks that treat trauma as discrete, resolvable events. This research calls for critical awareness of Western frameworks’ limitations while actively incorporating Palestinian epistemologies. The goal is creating hybrid approaches that honor Palestinian ways of knowing without Western domination. Ultimately, this study contributes to decolonial scholarship serving Palestinian liberation, supporting struggles for narrative sovereignty while challenging Western complicity in ongoing settler-colonial violence.

## Data Availability

The original contributions presented in the study are included in the article/supplementary material, further inquiries can be directed to the corresponding author.
